# Brief communication: immunogenicity of measles vaccine when co-administered with 10-valent pneumococcal conjugate vaccine

**DOI:** 10.1038/s41541-020-00225-z

**Published:** 2020-08-18

**Authors:** Zheng Quan Toh, Beth Temple, Tran Ngoc Huu, Vo Thi Trang Dai, Nguyen Trong Toan, Doan Y. Uyen, Kathryn Bright, Lien Anh Ha Do, E. Kim Mulholland, Paul V. Licciardi

**Affiliations:** 1 New Vaccines, Murdoch Children’s Research Institute, Royal Children’s Hospital, Parkville, VIC Australia; 2 Department of Paediatrics, The University of Melbourne, Parkville, VIC Australia; 3 Global and Tropical Health Division, Menzies School of Health Research, Charles Darwin University, Darwin, NT Australia; 4 Department of Infectious Disease and Epidemiology, London School of Hygiene and Tropical Medicine, London, UK; 5 Microbiology and Immunology, Pasteur Institute of Ho Chi Minh City, Ho Chi Minh City, Vietnam; 6 Department of Disease Control and Prevention, Pasteur Institute of Ho Chi Minh City, Ho Chi Minh City, Vietnam

**Keywords:** Vaccines, Public health, Translational research

## Abstract

This brief communication describes the findings from a randomised controlled trial in Vietnam that co-administration of measles vaccine (MV) with 10-valent pneumococcal conjugate vaccine (PCV10, Synflorix®, GSK) does not affect the immunogenicity of MV. These findings are most relevant for low- and middle-income countries (LMICs) in Asia considering PCV introduction.

## Introduction

Measles is one of the most highly contagious human pathogens, accounting for significant morbidity and mortality worldwide, with the highest burden in low- and lower-middle-income countries^1^. Routine measles vaccination for children, combined with mass immunisation campaigns in countries with low routine coverage, are key public health strategies to reduce global measles deaths. However, the global vaccine coverage of two-dose measles vaccine (MV) schedules (85 and 67% for first and second dose, respectively) are well short of the recommended 95% coverage needed to prevent outbreaks^2^. The recent global measles outbreak has claimed more than 140,000 lives, mostly among young children in the poorest countries^2^. In the first three months of 2019, provisional data based on monthly reports to the World Health Organization (WHO; as of April 2019) showed an almost 300% increase in the number of measles cases reported globally compared with the same period in 2018^3^. Such outbreaks highlight the importance of immunisation and the maintenance of high MV coverage in the country.

The Expanded Programme on Immunization (EPI) was established in 1974 to improve vaccination rates against measles, diphtheria, pertussis, tetanus, poliomyelitis and tuberculosis. Since then many other vaccines have been added to the EPI schedule, including Hepatitis B, yellow fever and *Haemophilus influenzae* (Hib) conjugate vaccine, and more recently pneumococcal conjugate vaccines (PCV) and rotavirus vaccine. Some of these vaccines are given simultaneously (co-administered) to alleviate logistical constraints related to vaccine delivery and also improve vaccine coverage. The exact EPI schedule varies by country.

PCV protects against S*treptococcus pneumoniae* infection, a leading cause of pneumonia and serious invasive diseases in children under 5 years^4^. Two licensed PCVs, 10-valent (PCV10, Synflorix; GSK) and 13-valent PCV (PCV13, Prevnar-13/Prevenar-13; Pfizer), are available and are demonstrated to be safe and effective in preventing vaccine-type pneumococcal diseases^5^. While these vaccines are in use in many regions, PCV introduction in Asia has been slow, in part due to the high vaccine cost, and also a lack of local or regional data on the effect of PCV for the government to consider PCV introduction. This is a priority as Asia, in particular the South East Asia region has one of the highest burden of pneumococcal disease globally with approximately 4.4 million cases occurring each year^4^. One important consideration for countries planning to introduce PCV is whether it will interfere with the immunogenicity of other EPI vaccines. WHO recommends PCV to be given as a three-dose schedule, either as a two-dose primary series followed by a booster dose at 9–18 months (2 + 1) or as a three-dose primary series without a booster dose (3 + 0), for maximum efficacy. These schedules coincide with existing EPI vaccines and so it is necessary to ensure that their addition does not interfere with the immunogenicity and protection of any co-administered vaccines.

In Vietnam, measles vaccination is given as two-dose schedule under the EPI; MV (AIK-C strain, POLYVAC, Vietnam) at 9 months of age and measles–rubella (MR) combination vaccine at 18 months of age. As a booster dose of PCV may also be given at 9 months of age, it is important to determine whether this has any unintended consequences on MV immunogenicity. As a secondary analysis within a randomised, single-blind controlled trial of alternative PCV schedules in Vietnam (the Vietnam Pneumococcal Project)^6^, we investigated whether co-administration of PCV10 with MV at 9 months of age interferes with the immunogenicity of MV.

## Results and discussion

**Fig. 1:**
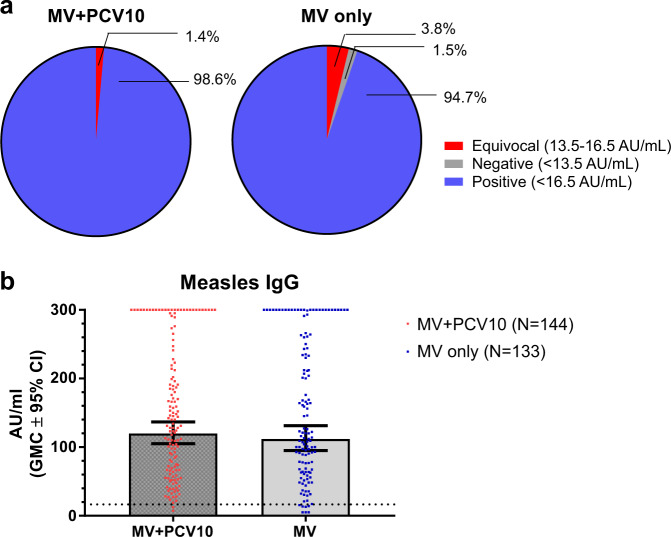
Measles IgG responses at 10 months of age; 1 month after a dose of measles vaccine (MV) at 9 months of age. **a** Proportions of positive, negative or equivocal measles IgG responses and **b** concentration of measles IgG responses. Dotted line represents 175 mIU/mL based on the WHO Third International Standard for Anti-Measles. PCV10 10-valent pneumococcal conjugate vaccine. Data presented as GMC ± 95% CI. GMC geometric mean concentrations, CI confidence interval.

Blood samples were collected at 10 months of age, from 144 participants who received both MV and PCV10 and from 133 participants who received MV alone at 9 months of age. The majority of participants in both groups were seropositive for measles antibody response; 142 (98.6%, 95% CI: 95.1–99.8%) in the MV-PCV10 group and 126 (94.7%, 95% CI: 89.5–97.9%) in the MV-only group (Fig. 1a). Both groups had similar measles IgG geometric mean concentrations (GMCs) [GMC 95% CI: MV-PCV10: 120 AU/mL (105.2–136.9) MV-only: 111.8 AU/mL (95.2–131.4), *P* = 0.502] (Fig. 1b).

This is the first study in Asia to evaluate the co-administration of PCV and MV. Our data demonstrate that co-administration of PCV10 and MV at 9 months of age does not affect the immunogenicity of MV. In addition, the reporting rates of adverse events were very low, following administration of either MV and PCV10 or MV alone. Only 5/143 (3.5%, data not available for one participant) people in the MV-PCV10 group and 2/133(1.5%) in the MV-only group reported any fever, with no other adverse events reported. The results of this study provide evidence for the Vietnamese government and other countries in the region to support the introduction of PCV10 in a schedule in which the booster dose coincides with the first dose of MV.

PCV10 co-administration has previously been reported not to impact on the immunogenicity or reactogenicity of other childhood vaccines such as Infanrix™-IPV/Hib (diphtheria–tetanus–acellular pertussis-inactivated poliovirus–*H. influenzae* type b) or Infanrix™ hexa (Infanrix™-IPV/Hib with hepatitis B antigens), and Rotarix™ (live attenuated human rotavirus vaccine)^7–9^. Data on the co-administration of PCV and MV are limited, particularly for Asian countries. A multi-site trial in Europe examined the co-administration of PCV10 with a tetravalent vaccine containing measles–mumps–rubella–varicella (MMRV, Priorix-Tetra^TM^, GSK Biologicals)^10^. The seroconversion rates for measles were over 97% following co-administration with either PCV10 or Infanrix™-hexa, with similar measles antibody levels between both groups^10^. However, there was not a group that received only MMRV for comparison. A recent trial of PCV formulations containing pneumococcal proteins in The Gambia administered MV at 9 months of age (along with yellow fever and oral polio vaccines), and included groups that either received PCV10 in a 2 + 1 schedule (with the booster dose co-administered with MV; M-Vac, Serum Institute of India) or a 3 + 0 schedule^11^. Three months post-vaccination, the proportion of measles antibody levels equal to or above the seroprotective level were not statistically different between the children in the 3 + 0 group [72.9% (95% CI: 62.9–81.5)] and 2 + 1 group [84.0% (95% CI 75.0–90.8) in the 2 + 1 group]. Measles GMC antibody levels and reactogenicity (injection site pain being the most frequent, between 5 and 10%) were similar in both groups. Although a different MV strain and population were evaluated in this study compared to our study, the findings were consistent in that no immune interference and no differences in reactogenicity when PCV10 were co-administered with MV was observed.

In conclusion, in line with other studies, we have demonstrated a high measles seroconversion rate among children who received both PCV10 and MV. Our results therefore support the co-administration of PCV10 and MV at 9 months of age. These findings are most relevant for LMICs in Asia that are considering PCV introduction.

## Methods

### Study design and participants

Two groups within the Vietnam Pneumococcal Project (ClinicalTrials.gov, number NCT01953510) received PCV10 in either a 3 + 1 schedule (at 2, 3, 4 and 9 months of age), or a 3 + 0 schedule (at 2, 3 and 4 months of age). Both groups received a first dose of MV at 9 months of age. Blood samples were collected 4 weeks post-MV. Details of the study design have been published previously^6^. All relevant ethical regulations for work with human participants have been adhered; ethical approval has been obtained from the Human Research Ethics Committee of the Northern Territory Department of Health and Menzies School of Health Research (EC00153) and the Vietnam Ministry of Health Ethics Committee and written informed consent from the parent/legal guardian were obtained.

### Measurement of measles IgG response

The MV IgG response was examined using a commercial chemiluminescence immunoassay (Diasorin, USA). The assay detection range is 5–300 AU/mL. The criteria for classifying MV IgG responses were: negative: <13.5 AU/mL, equivocal: 13.5–16.5 AU/mL and positive: ≥16.5 AU/mL. The positive cut-off value equates to 175 mIU/mL based on the WHO Third International Standard for Anti-Measles; a titre of ≥120 mIU/mL by plaque reduction neutralisation test (PRNT) is considered protective. A previous study comparing this commercial immunoassay with PRNT has found that samples in the equivocal range were >120 mIU/mL^12^. The MV IgG GMCs were log-transformed and compared using Student’s *t*-test.

### Reporting summary

Further information on research design is available in the Nature Research Reporting Summary linked to this article.

## Supplementary information

Reporting Summary

## Data Availability

The datasets generated during and/or analysed during the current study are available from the corresponding author on reasonable request.
