# Supplementary material for: Brief communication: immunogenicity of measles vaccine when co-administered with 10-valent pneumococcal conjugate vaccine
Source: NPJ Vaccines. 2020 Aug 18;5:76. doi: 10.1038/s41541-020-00225-z (PMC7434759; doi:10.1038/s41541-020-00225-z)
Supplement: Supplementary file 1 — Reporting Summary [file 41541_2020_225_MOESM1_ESM.pdf]

nature research

Corresponding author(s): Paul V Licciardi

Last updated by author(s): Jun 30, 2020

Reporting Summary

Nature Research wishes to improve the reproducibility of the work that we publish. This form provides structure for consistency and transparency in reporting. For further information on Nature Research policies, see our Editorial Policies and the Editorial Policy Checklist.

Statistics

For all statistical analyses, confirm that the following items are present in the figure legend, table legend, main text, or Methods section.

n/a	Confirmed	
☐	☒	The exact sample size (*n*) for each experimental group/condition, given as a discrete number and unit of measurement
☐	☒	A statement on whether measurements were taken from distinct samples or whether the same sample was measured repeatedly
☐	☒	The statistical test(s) used AND whether they are one- or two-sided<br>*Only common tests should be described solely by name; describe more complex techniques in the Methods section.*
☒	☐	A description of all covariates tested
☒	☐	A description of any assumptions or corrections, such as tests of normality and adjustment for multiple comparisons
☐	☒	A full description of the statistical parameters including central tendency (e.g. means) or other basic estimates (e.g. regression coefficient) AND variation (e.g. standard deviation) or associated estimates of uncertainty (e.g. confidence intervals)
☒	☐	For null hypothesis testing, the test statistic (e.g. *F*, *t*, *r*) with confidence intervals, effect sizes, degrees of freedom and *P* value noted<br>*Give P values as exact values whenever suitable.*
☒	☐	For Bayesian analysis, information on the choice of priors and Markov chain Monte Carlo settings
☒	☐	For hierarchical and complex designs, identification of the appropriate level for tests and full reporting of outcomes
☒	☐	Estimates of effect sizes (e.g. Cohen's *d*, Pearson's *r*), indicating how they were calculated

Our web collection on statistics for biologists contains articles on many of the points above.

Software and code

Policy information about availability of computer code

Data collection	No software was used
Data analysis	Graphpad Prism

For manuscripts utilizing custom algorithms or software that are central to the research but not yet described in published literature, software must be made available to editors and reviewers. We strongly encourage code deposition in a community repository (e.g. GitHub). See the Nature Research guidelines for submitting code & software for further information.

Data

Policy information about availability of data

All manuscripts must include a data availability statement. This statement should provide the following information, where applicable:

- Accession codes, unique identifiers, or web links for publicly available datasets
- A list of figures that have associated raw data
- A description of any restrictions on data availability

The datasets generated during and/or analysed during the current study are available from the corresponding author on reasonable request

Field-specific reporting

Please select the one below that is the best fit for your research. If you are not sure, read the appropriate sections before making your selection.

☒ Life sciences ☐ Behavioural & social sciences ☐ Ecological, evolutionary & environmental sciences

For a reference copy of the document with all sections, see nature.com/documents/nr-reporting-summary-flat.pdf

Life sciences study design

All studies must disclose on these points even when the disclosure is negative.

Sample size	This study was part of a secondary analysis within a randomised, single-blind controlled trial of alternative PCV schedules in Vietnam (the Vietnam Pneumococcal Project). Sample size from each group was determined based on the primary outcome.
Data exclusions	No data was excluded
Replication	All experimental procedures were performed with relevant controls and the results were validated.
Randomization	This study was part of a secondary analysis within a randomised ,single-blind controlled trial. The groups were allocated based on the primary outcome. Allocation sequence was produced using a computer-generated list of random numbers using a block randomisation scheme.
Blinding	All laboratory staff are blinded to the study group allocation

Reporting for specific materials, systems and methods

We require information from authors about some types of materials, experimental systems and methods used in many studies. Here, indicate whether each material, system or method listed is relevant to your study. If you are not sure if a list item applies to your research, read the appropriate section before selecting a response.

Materials & experimental systems

n/a	Involved in the study	
☒	☐	Antibodies
☒	☐	Eukaryotic cell lines
☒	☐	Palaeontology and archaeology
☒	☐	Animals and other organisms
☐	☒	Human research participants
☐	☒	Clinical data
☒	☐	Dual use research of concern

Methods

n/a	Involved in the study	
☒	☐	ChIP-seq
☒	☐	Flow cytometry
☒	☐	MRI-based neuroimaging

Human research participants

Policy information about studies involving human research participants

Population characteristics	This study was part of a larger randomised controlled trial described above. Healthy infants from Ho Chi Minh City, VIetnam aged between 2 months and 2 months plus 2 weeks with no significant maternal or perinatal history; born at or after 36 weeks' gestation; written informed consent from the parent/legal guardian; lives within approximately 30min of the commune health centre; anticipates living in the study area for the next 22 months
Recruitment	This study was part of a larger randomised controlled trial described above. Participants are recruited from infants born in the study communes during the enrolment period. Commune health centre staff identify potential participants from the commune health centre birth records. Recruitment rates were monitored on a monthly basis and meetings held with study staff and commune health centre staff to discuss any significant declines in recruitment rates. Commune health centre staff visit the home of potential participants when the infant is approximately 6weeks old and provide verbal and written information about the trial, in Vietnamese. Those interested in participating are referred to the study clinic when the infant is approximately 2months old. At this time, written informed consent is obtained, after which a study nurse/doctor examines the infant to ensure that all the eligibility criteria are met.
Ethics oversight	The Institutional Review Board at the Pasteur Institute of Ho Chi Minh City, the Vietnam Ministry of Health Ethical Review Committee and the Human Research Ethics Committee of the Northern Territory Department of Health and the Menzies School of Health Research.

Note that full information on the approval of the study protocol must also be provided in the manuscript.

Clinical data

Policy information about clinical studies

All manuscripts should comply with the ICMJE guidelines for publication of clinical research and a completed CONSORT checklist must be included with all submissions.

Clinical trial registration	ClinicalTrials.gov, number NCT01953510
Study protocol	Temple B, Toan NT, Uyen DY, et alEvaluation of different infant vaccination schedules incorporating pneumococcal vaccination (The Vietnam Pneumococcal Project): protocol of a randomised controlled trialBMJ Open 2018;8:e019795. doi: 10.1136/ bmjopen-2017-019795
Data collection	Standardised carbon copy data collection forms are used and are completed by dedicated, trained study staff. The original is transported to the trial office for data entry, with the carbon copy filed at the clinic. Blood samples are collected by staff specifically trained in the collection of samples from infants, and the volume of blood collected are recorded. Blood samples collected into gel vacutainer tubes are kept chilled in a cooler box and transported to the Pasteur Institute laboratory the same day. On arrival at the laboratory the samples are centrifuged and the sera divided into up to three aliquots, stored in micro-tubes and frozen at -80°C prior to analysis.
Outcomes	This study is a secondary analysis within a randomised, single-blind controlled trial of alternative PCV schedules in Vietnam (the Vietnam Pneumococcal Project). The primary aim for this study is to determine whether co-administration of PCV10 with measles vaccine at 9 months of age interferes with the immunogenicity of measles vaccine. The primary outcome is the measles antibody response one month following measles vaccination. Blood samples collected 4 weeks post-measles vaccination from two study groups (received measles vaccine alone or measles and PCV10 simultaneously) was examined using a commercial chemiluminescence immunoassay (Diasorin, USA).
